# Changes of natural killer cells’ phenotype in patients with chronic hepatitis B in intermittent interferon therapy

**DOI:** 10.3389/fimmu.2023.1116689

**Published:** 2023-01-30

**Authors:** Xiaoyue Bi, Si Xie, Shuling Wu, Weihua Cao, Yanjie Lin, Liu Yang, Tingting Jiang, Wen Deng, Shiyu Wang, Ruyu Liu, Yuanjiao Gao, Ge Shen, Min Chang, Hongxiao Hao, Mengjiao Xu, Xiaoxue Chen, Leiping Hu, Yao Lu, Lu Zhang, Yao Xie, Minghui Li

**Affiliations:** ^1^ Department of Hepatology Division 2, Beijing Ditan Hospital, Capital Medical University, Beijing, China; ^2^ Division of Hepatology, Hepato-Pancreato-Biliary Center, Beijing Tsinghua Changgung Hospital, School of Clinical Medicine, Tsinghua University, Beijing, China; ^3^ Department of Infectious Diseases, Miyun Teaching Hospital, Capital Medical University, Beijing, China; ^4^ Department of Hepatology Division 2, Peking University Ditan Teaching Hospital, Beijing, China

**Keywords:** natural killer cell, chronic hepatitis B, antiviral treatment, intermittent therapy, PEG-IFN α

## Abstract

**Background:**

To investigate the changes of natural killer (NK) cell phenotype in the interferon alpha (IFN-α) treatment of chronic hepatitis B (CHB) and its relationship with clinical indicators.

**Methods:**

The CHB patients who did not receive any antiviral treatment were set as initial treatment group and used pegylated interferon alpha (PEG-IFN α). Peripheral blood samples were collected at baseline, 4 weeks, and 12-24 weeks. For IFN-treated patients who entered the plateau were set as plateau group, and PEG-IFN α was discontinued and resumed after an interval of 12-24 weeks. Besides, we also enrolled some patients who had received oral drug for more than 6 months as oral drug group without follow up. Peripheral blood was collected during the plateau period, which was set as baseline, and after 12-24 weeks of intermittent treatment, and after 12-24 weeks of additional treatment with PEG-IFN α. The aim of the collection was to detect hepatitis B virus (HBV) virology, serology and biochemical indicators, and the NK cell related phenotype was detected by flow cytometry.

**Results:**

In the plateau group, subgroup of CD69^+^CD56^dim^ was higher with statistical significance when comparing with the initial treatment group and oral drug group [10.49 (5.27, 19.07) vs 5.03 (3.67, 8.58), Z = -3.11, *P* = 0.002; 10.49 (5.27, 19.07) vs 4.04 (1.90, 7.26), Z = -5.30, *P* < 0.001)]. CD57^+^CD56^dim^ was significantly lower than that in initial treatment group and oral drug group respectively [68.42±10.37 vs 55.85±12.87, t = 5.84, *P* < 0.001; 76.38±9.49 vs 55.85±12.87, t = -9.65, *P* < 0.001]. The CD56^bright^CD16^-^ subgroup in the plateau group was higher with statistical significance compared with initial treatment group and oral drug group respectively [11.64 (6.05, 19.61) vs 3.58 (1.94, 5.60), Z = -6.35, *P* < 0.001; 11.64 (6.05, 19.61) vs 2.37 (1.70, 4.30), Z = -7.74, *P* < 0.001)]. CD57^+^CD56^dim^ in the plateau group had a significant higher percentage than that at baseline after IFN discontinuation for 12-24 weeks (55.85±12.87 vs 65.95±12.94, t = -2.78, *P* = 0.011).

**Conclusion:**

During the long-term treatment of IFN, the killer subgroup of NK cells is continuously depleted, leading to the differentiation of the regulatory subgroup into the killer subgroup. In the killing subgroup, although the number is continuously depleted, the activity of the subgroup is continuously increased. In the plateau phase, after stopping IFN for a period of time, the number of NK cell subsets would gradually recover, but was still lower than that in the initial treatment group.

## Introduction

At present, about 257 million people are suffered from HBV infection and according to a survey in 2015, there was a number of 887 thousand deaths happened because of HBV infection-related diseases, including liver cirrhosis and hepatocellular carcinoma (HCC), which comprised about 52% and 38% respectively (1). It is of great necessity for CHB patients to receive timely therapy because of the huge threat and damage brought by this disease. One method with great effectiveness to decrease the risk of HCC is eliminating hepatitis B surface antigen (HBsAg), which is also an indicator attached with great importance to test the long-term prognosis of CHB patients, as well a key result of functional cure (2, 3). Yeo et al. found in a recent meta-analysis that in CHB patients without any antiviral treatments, the disappearance rate of HBsAg was only 1.02% yearly. Based on the above researches, antiviral treatment is considered to be necessary for CHB patients (4).

The recommended antiviral drugs in guidelines are nucleoside (nucleotide) analogues (NAs) and IFN-α. In the researches we have conducted before, the disappearance rate of HBsAg could maintain at 15% in CHB patients treated by PEG-IFN α combined therapy or extended therapy, which was better than using NAs alone (5). However, in the process of HBsAg elimination, it is not achieved easily through one-time combined therapy or extended therapy, but through multiple stages, which is called intermittent therapy. When received long-term IFN treatment, some patients’ HBsAg decline level may come to a plateau, which is defined as HBsAg level decreases < 0.5lg IU/ml compared to last detection. Even continuing with previous IFN treatment in plateau period, it is not easy to achieve sustained virological response for patients. Meanwhile, it is also recommended in guidelines that if the decrease level of HBsAg < 1lg IU/ml after 24-week IFN therapy, the antiviral therapy should be replaced by NAs (6, 7). Therefore, for patients stagging in plateau period, we conducted intermittent therapy for them. About 20% of the patients achieved HBsAg disappearance through this mode and no HBsAg disappearance happened in the patients who stopped interferon in the plateau period and did not receive interferon re-treatment (8). This indicates that interferon intermittent therapy has an ideal outlook that can improve the HBsAg conversion rate in patients with CHB. However, the molecular mechanism of intermittent therapy and the causes of the plateau need to be further explored.

NK cells can be defined as an crucial component of antiviral immune response and can eliminate infected cells through cytotoxicity or other mechanisms (9). The role of NK cells in chronic HBV infection has also been reported, saying that HBV infection may alter the activation state of NK cells and the expression of relevant receptors on the cell surface (10). A research about CHB patients’ cessation of long-term nucleoside analogue therapy showed that HBsAg clearance was closely related to NK cells’ phenotype and function (11). In patients treated with IFN, long-term exposure to IFN-α can increase the expression level of STAT1, promote its phosphorylation and activation, and lead to phenotypic changes in NK cells and increased cytotoxicity (12, 13). In CHB patients who gained functional cure through IFN therapy, dynamic changes of NK cells were also associated with HBsAg disappearance (14). In line with the above studies, we believe that the changes and variety of NK cell phenotype during intermittent treatment are worth exploring.

## Materials and methods

### Patients

Patients diagnosed as CHB by Hepatology Center of Beijing Ditan Hospital from November 2021 to August 2022 were enrolled as subjects and were divided into three subgroups: initial treatment group, Plateau group, and oral drug group. CHB was defined as HBsAg positive ≥ 24 weeks, with or without hepatitis B e antigen (HBeAg) positive, and abnormal ALT (> 120U/L) more than 12 weeks. Patients who had never received any treatment before and began to use IFN at the first time were initial treatment group; who had received first-line oral dug more than 6 months were oral drug group; and who had used IFN for a period then the level of HBsAg decreased < 0.5lg IU/ml were plateau group. Exclusion criteria: 1) combined-infection with other viral, such as hepatitis C virus (HCV), hepatitis D virus (HDV), hepatitis E virus (15), etc.; 2) long-term use of immunosuppressive drugs or drugs that can easily cause liver damage; 3) other diseases which are liver-related, such as alcoholic hepatitis, autoimmune hepatitis, metabolic liver disease, cirrhosis, liver tumors, etc.; 4) mental illness, such as depression; 5) serious cardiovascular and cerebrovascular diseases or other systemic diseases with greater harm.

The oral drug group was set up to make a cross-sectional comparison with the initial treatment group and the plateau group, aiming to explore the regulatory effects of oral drug and PEG-IFN α treatment on NK cells. Meanwhile, the results also showed that the percentages of NK cell modulation subsets and killing subsets did not change significantly after oral drug treatment compared with the initial treatment group. To further investigate the regulation of NK cell subsets by oral medication, regular follow-up should be made. However, this study mainly discussed the changes of NK cell subgroup during interferon treatment, so the dynamic changes during follow up in oral group was not included in the study.

Setting up corresponding control group (IFN treatment continued after HBsAg plateau) can better reflect the advantages of intermittent therapy and the relationship between NK cells and treatment strategy. However, according to the Chinese Guidelines for the Prevention and Treatment of Chronic Hepatitis B (2019 edition) (1), IFN should be discontinued after 24 weeks of PEG-IFN α treatment when HBsAg drops to < 1lgIU/ml. Therefore, in this study, we did not establish such a control group.

This was a prospective study approved by the Ethics Committee of Beijing Ditan Hospital (Jing Di Lun Ke Zi 2018 no. 023-01), with registration of Clinical Trial (NCT04028856). All subjects have signed informed consent before enrollment.

### Sample collection

**Figure 1:**
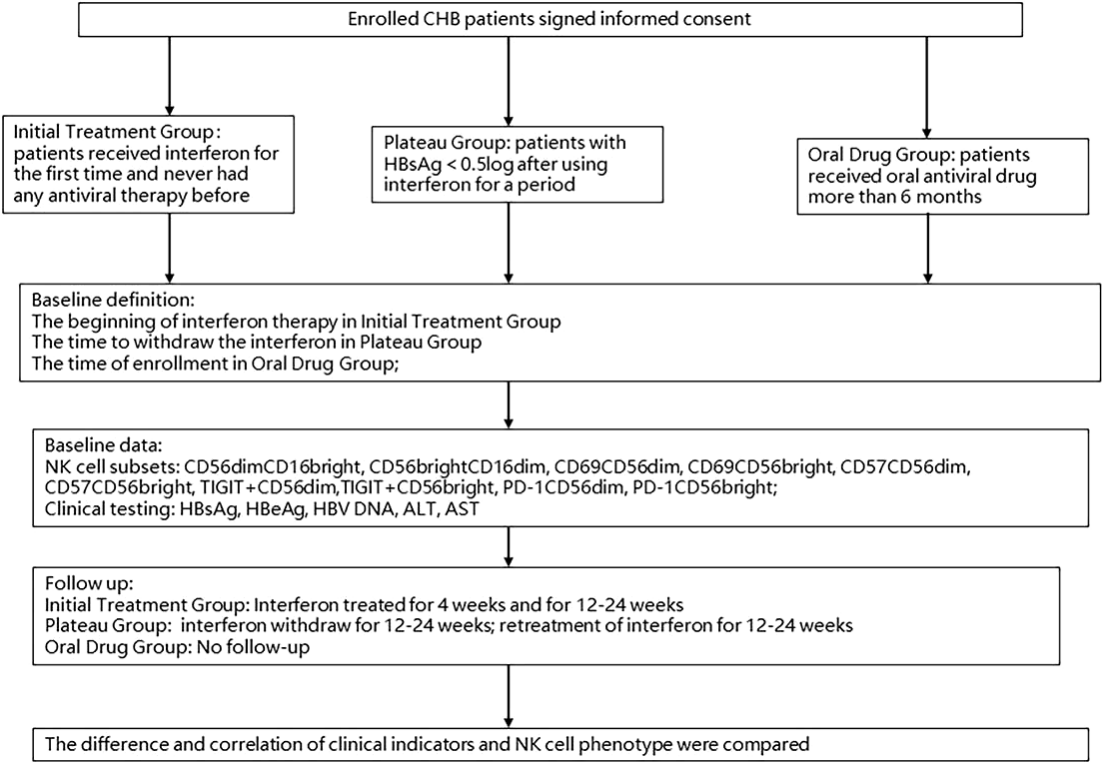
Procession of enrollment.

In the initial treatment group, patients began to receive subcutaneous injection of PEG-IFN α-2a 180 μg weekly, combined with first-line antiviral drugs (entecavir [ETV] 0.5 mg/d or tenofovir disoproxil fumarate [TDF] 300 mg/d or tenofovir alafenamide fumarate [TAF] 25 mg/d) according to their clinical characteristic and personal willing. The beginning time of IFN-α was set as baseline and the 4^th^ week and the 12rd to 24^th^ week were follow-up points. The plateau group was based on interferon discontinuation at baseline and followed up 12 to 24 weeks after discontinuation and 12 to 24 weeks after re-introduction. During the intermittent, first-line antiviral drugs (ETV 0.5 mg/d or TDF 300 mg/d or tenofovir alafenamide fumarate [TAF] 25 mg/d) were applied to maintain antiviral effect. In the oral drug group, baseline was set as the time of enrollment and no need to follow up. At baseline and each follow-up time point, we used two EDTA purple tubes with a capacity of 9 ml respectively to collect peripheral venous blood to test clinical indicators such as HBsAg, HBeAg, HBV DNA and liver function. Peripheral blood mononuclear cell (PBMC) determination was extracted within 4 hours of peripheral blood collection for subsequent staining and flow cytometry. The process of enrollment and detection is shown in **Figure 1**.

### Peripheral blood NK cell phenotype

**Figure 2:**
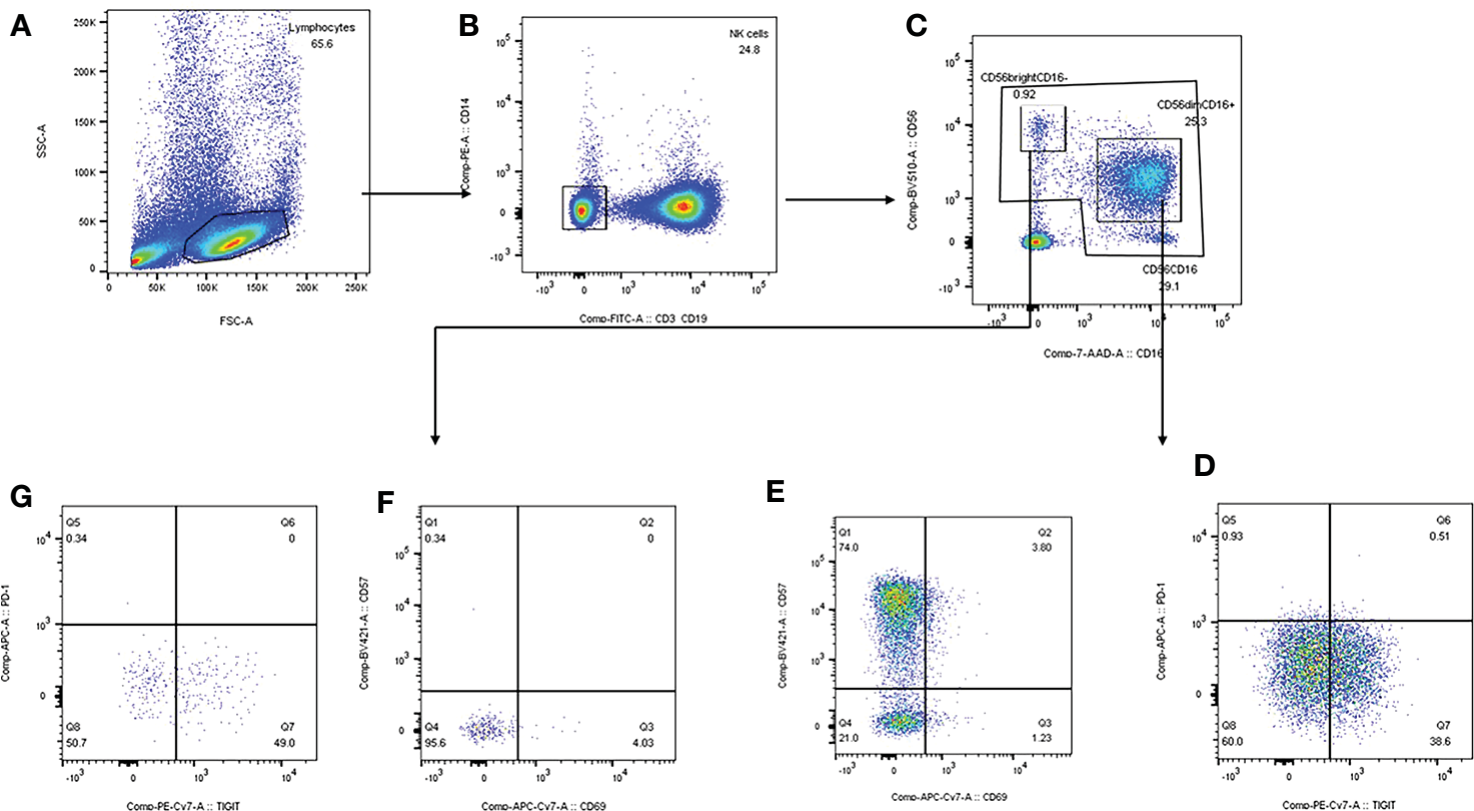
Strategy of subgroup gate and analyzation on FlowJo software. **(A)** mononuclear cells were separated according to side scatter and forward scatter. **(B)** We draw the gate of NK cells based on CD3/CD19-FITC and CD14-PE. **(C)** CD56^dim^CD16^+^ and CD56^bright^CD16^-^ were separated by CD16-7-AAD and CD56-BV510. **(D, E)** CD69^+^CD56^dim^, CD57^+^CD56^dim^, TIGIT^+^CD56^dim^, PD-1^+^CD56^dim^ were delineated by TIGIT-PE-Cy7, PD-1-APC, CD69-APC-Cy7 and CD57-BV421. **(F, G)** CD69^+^CD56^bright^, CD57^+^CD56^bright^, TIGIT^+^CD56^bright^, PD-1^+^CD56^bright^ were delineated by TIGIT-PE-Cy7, PD-1-APC, CD69-APC-Cy7 and CD57-BV421.

The PBMC was extracted and resuspended with phosphate buffered saline (PBS). Anti-human antibodies CD3/CD19-Fluorescein Isothiocyanate (FITC), CD14-phycoerythrin (PE) anti-human antibody, CD16-peridinin green protein (PerCP) anti-human antibody, CD56-Brilliant Violet 510 (BV510) anti-human antibody, CD57-BV421 anti-human antibody, CD69-allophycocyanin (APC) anti-human antibody, TIGIT-PE anti-human antibody, PD-1-APC anti-human antibody were added to the 100μl PBMC cell suspension sequently according to instructions. After full vortex mixing, leave samples avoid light at room temperature for 15-20min to dye. After staining, 2ml PBS was added, and after 1200g×5min centrifugation, the supernatant was discarded, and 200μl PBS was added to homogenize the cell mass at the bottom of the tube. Canto flow cytometry was used for detection. Voltage and fluorescence compensation were adjusted to the optimum, cells were collected, and data were derived for ringgate analysis using Flowjo software. The strategy of subgroup gate is shown in **Figure 2**.

### Detection of clinical indicators

HBsAg and HBeAg were detected by chemiluminescent particle method (Abbott Diagnostics, Abbott Park, IL, USA). If the concentration of HBsAg was larger than 250U/ml, the sample was diluted to 1:500 and the specific concentration was calculated. Real-time quantitative polymerase chain reaction (PCR) was used to detect HBV DNA concentration in serum (Piji Co, Ltd, Shenzhen, China). ALT an AST were detected by Wako Pure Chemical Industries, Ltd., Japan.). All clinical indicators were tested by Ditan Hospital Inspection Center.

### Statistical analysis

All data were analyzed by SPSS25.0 (IBM Corporation, Chicago, IL, USA). The measurement data were first tested by Shapiro-Wilktest for normality. Those meeting the normality test were represented by mean ± standard deviation, while those not meeting the normality distribution were represented by median (Q1, Q3). Data were compared between groups by t test, Wilcoxon test or Mann-Whitney test. Linear regression was used to analyze the correlation between subsets of NK cells and clinical indicators (ALT, AST, HBsAg, HBsAg, HBV DNA). If *P* < 0.05, it was considered statistically significant. For repeated measurements, α segmentation method was used, and we set *P* < 0.017 as significant statistically.

## Result

### Baseline clinical features and NK cell phenotype of patients

176 patients with CHB were enrolled totally, including 51 in the initial treatment group, 50 in the plateau group and 75 in the oral drug group. The mean age was 34.24 (31.22, 41.16) years in initial treatment group, 34.24 (31.22, 41.16) years in plateau group, 48.00 (41.13, 58.01) in oral drug group. Between initial treatment group and oral drug group, CD56^dim^CD16^+^ subgroup [80.90 (69.41, 86.40) vs 79.30 (66.70, 84.82), Z = -1.03, *P* = 0.305] and CD56^bright^CD16^-^ subgroup [3.58 (1.94, 5.60) vs 2.37 (1.70, 4.30), Z = 11.78, *P* = 0.075)] had no significant differences, but when compared with the plateau group, the result was significantly different [CD56^dim^CD16^+^: 80.90 (69.41, 86.40) vs 46.51±18.51, Z = -7.08, *P* < 0.001 (initial treatment group vs plateau group); 79.30 (66.70, 84.82) vs 46.51±18.51, Z = -6.44, *P* < 0.001 (oral drug group vs plateau group); CD56^bight^CD16^-^: 3.58 (1.94, 5.60) vs 11.64 (6.05, 19.61), Z = -6.35, *P* < 0.001 (initial treatment group vs plateau group); 2.37 (1.70, 4.30) vs 11.64 (6.05, 19.61), Z = -7.74, *P* < 0.001 (oral drug group vs plateau group)]. When comparing the subgroup of CD69^+^CD56^dim^ in plateau group, initial treatment group and oral drug group, the former was significantly higher than the two others [10.49 (5.27, 19.07) vs 5.03 (3.67, 8.58), Z = -3.11, *P* = 0.002; 10.49 (5.27, 19.07) vs 4.04 (1.90, 7.26), Z = -5.30, *P* < 0.001], while CD57^+^CD56^dim^ was significantly lower than that in initial treatment group and oral drug group [68.42±10.37 vs 55.85±12.87, t = 5.84, *P* < 0.001; 76.38±9.49 vs 55.85±12.87, t = -9.65, *P* < 0.001]. The expression of CD56^bright^CD16^-^ in plateau group was significantly higher than that in initial treatment group and oral administration group [11.64 (6.05, 19.61) vs 3.58 (1.94, 5.60), Z = -6.35, *P* < 0.001; 11.64 (6.05, 19.61) vs 2.37 (1.70, 4.30), Z = -7.74, *P* < 0.001]. The baseline characteristic was shown in **Table 1**.

**Table 1:** Characteristics of initial treatment group, plateau group and oral drug group at baseline.

	Initial treatment group(n=51)	Plateau group(n=50)	Oral drug group(n=75)	Z/t			P		
				C0 vs P0	C0 vs N0	P0 vs N0	C0 vs P0	C0 vs N0	P0 vs N0
**Age (years)** **(median[Q1,Q3])**	34.24(31.22, 41.16)	36.17(32.88, 44.04)	48.00(41.13, 58.01)	-1.519	-4.97	-3.99	0.129	<0.001	<0.001
**Gender(male/female)**	31/20	19/31	27/48	5.24	7.51	0.05	0.022	0.006	0.820
**HBsAg (log10IU/ml)**	3.80(3.28, 4.25)	2.62(0.96, 3.42)	2.94(2.24, 3.57)	-4.57	-3.68	-1.74	<0.001	<0.001	0.082
**HBeAg (S/CO)**	15.86(0.35, 1256.44)	0.39(0.32, 4.90)	0.53(0.40, 29.31)	-3.231	-1.47	-1.92	0.001	0.142	0.055
**HBV DNA (log10IU/ml)**	4.53(2.28, 8.11)	1.00(0.04, 1.00)	0.52(0.04, 1.00)	-6.22	-5.69	-1.50	<0.001	<0.001	0.133
**ALT (U/L)**	35.00(25.38, 77.98)	34.00(25.60, 47.80)	25.50(20.75, 39.50)	-0.861	-2.63	-2.26	0.389	0.009	0.024
**AST (U/L)**	27.05(18.78, 64.80)	29.60(22.60, 39.50)	23.00(19.00, 27.50)	-0.01	-1.92	-3.15	0.992	0.055	0.002
**CD56^dim^CD16^+^**	80.90(69.41,86.40)	46.51±18.51	79.30(66.70,84.82)	-7.08	-1.03	-6.44	<0.001	0.305	<0.001
**CD69^+^CD56^dim^**	5.03(3.67, 8.58)	10.49(5.27, 19.07)	4.04 (1.90, 7.26)	-3.11	-2.43	-5.30	0.002	0.015	<0.001
**CD57^+^CD56^dim^**	68.42±10.37	55.85±12.87	76.38±9.49	5.84	-3.88	-9.65	<0.001	<0.001	<0.001
**TIGIT^+^CD56^dim^**	49.94±21.19	43.2(30.8, 56.1)	44.71±19.48	-0.60	1.42	-0.64	0.423	0.157	0.521
**PD-1^+^CD56^dim^**	1.97(0.964, 3.9)	1.5(0.728, 5.16)	0.39 (0.17, 0.71)	-0.65	-6.54	-5.59	0.548	<0.001	<0.001
**CD56^bright^CD16^-^**	3.58(1.94,5.60)	11.64(6.05,19.61)	2.37(1.70,4.30)	-6.35	-1.78	-7.74	<0.001	0.075	<0.001
**CD69^+^CD56^bright^**	3.10(1.95, 5.07)	2.97(2.14, 4.2)	1.57 (0.00, 2.24)	-0.11	-4.70	-4.81	0.913	<0.001	<0.001
**CD57^+^CD56^bright^**	0.48(0.00, 1.41)	0.51(0.24, 1.16)	1.56 (0.42, 3.96)	-0.61	-3.21	-2.81	0.545	0.001	0.005
**TIGIT^+^CD56^bright^**	8.10(5.79, 12.3)	9.04(6.72, 14.8)	7.67 (3.20, 30.71)	-1.20	-0.26	-0.95	0.232	0.794	0.343
**PD-1^+^CD56^bright^**	0.24(0.00, 0.79)	0.033(0, 0.991)	0.00 (0.00, 0.00)	-0.34	-4.10	-3.68	0.735	0.001	<0.001

HBsAg, hepatitis B surface antigen; HBeAg, hepatitis B e antigen; HBV DNA, hepatitis B virus deoxyribonucleic acid; ALT, alanine transaminase; AST, aspartate aminotransferase.

### Changes of NK cell phenotype during treatment

In the initial treatment group, CD56^dim^CD16^+^ subgroup, CD57^+^CD56^dim^ and TIGIT^+^CD56^dim^ all decreased gradually with the extension of treatment time, and the differences were statistically significant compared with baseline, respectively [80.90 (69.41, 86.40) vs 51.18 (34.34, 60.00). Z = -3.81, *P* < 0.001; 68.42±10.37 vs 57.14±10.92, t = 3.74, *P* = 0.001; 49.94±21.19 vs 30.04±14.22, t = 3.29, *P* = 0.004]. The expression of CD56^bright^CD16^-^ subgroup increased with the extension of treatment time, and the difference was statistically significant compared with baseline [3.58 (1.94,5.60 vs 10.70±5.95, Z = -3.17, *P* = 0.002].

**Figure 3:**
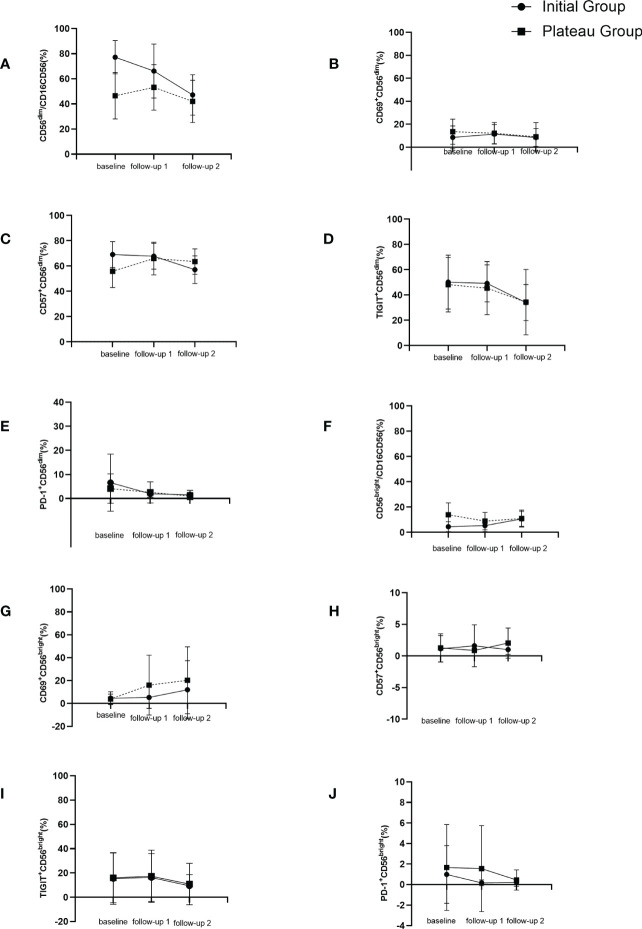
Dynamic changes of NK cell subgroups with follow-up time. **(A)** changes of CD56^dim^CD16^+^ (%) during follow up. **(B)** changes of CD69^+^CD56^dim^ (%) during follow up. **(C)** changes of CD57^+^CD56^dim^ (%) during follow up. **(D)** changes of TIGIT^+^CD56^dim^ (%) during follow up. **(E)** changes of PD-1^+^CD56^dim^ (%) during follow up. **(F)** changes of CD56^bright^CD16- (%) during follow up. **(G)** changes of CD69^+^CD56^bright^ (%) during follow up. **(H)** changes of CD57^+^CD56^bright^ (%) during follow up. **(I)** changes of TIGIT^+^CD56^bright^ (%) during follow up. **(J)** changes of PD-1^+^CD56^bright^ (%) during follow up.

As for plateau group, CD57^+^CD56^dim^ expression increased significantly higher after interferon discontinuation for 12-24 weeks compared to baseline (55.85±12.87 vs 65.95±12.94, t = -2.78, *P* = 0.011). There was no significant difference between baseline and untreated group (65.95±12.94 vs 68.42±10.37, t = 1.12, *P* = 0.266). The dynamic changes of NK cells’ subsets were shown in **Table 2** and **Figure 3**.

**Table 2:** Comparison of initial treatment group and plateau group during follow up.

	Initial Treatment Group									Plateau Group								
	W_0_(n=51)	W_4_(n=29)	W_12-24_(n=20)	Z/t			P			W_0_ (n=51)	Withdraw 12-24w (n=)	Retreat 12-24w (n=)	Z/t			P		
				W_0_vs W_4_	W_0_vsW_12-24_	W_4_ vs W_12-24_	W_0_vs W_4_	W_0_ vs W_12-24_	W_4_ vs W_12-24_				W0 vs Withdraw	W0 vs Retreat	Withdraw vs Retreat	W0 vs Withdraw	W0 vs Retreat	Withdraw vs Retreat
**CD56^dim^CD16^+^**	80.90(69.41,86.40)	71.58(63.19, 77.34)	51.18(34.34, 60.00)	-1.44	-3.81	-2.69	0.150	<0.001	0.007	0.4651±0.1851	0.5322±0.1807	5.63(1.30,14.60)	2.11	-1.35	-1.25	0.047	0.177	0.210
**CD69^+^CD56^dim^**	5.03(3.67, 8.58)	9.1(6.12,11.65)	4.99(2.99,10.68)	-2.00	-0.075	-1.09	0.045	0.940	0.278	10.49(5.27, 19.07)	10.68(4.07,17.30)	5.97(3.16,11.27)	-0.85	-0.83	-0.78	0.398	0.407	0.435
**CD57^+^CD56^dim^**	68.42±10.37	67.80±10.30	57.14±10.92	-1.02	3.74	3.63	0.315	0.001	0.002	55.85±12.87	65.95±12.94	63.58±10.14	-2.78	-2.37	-0.36	0.011	0.031	0.722
**TIGIT^+^CD56^dim^**	49.94±21.19	49.23±14.57	30.04±14.22	0.66	3.29	2.86	0.516	0.004	0.012	43.2(30.8, 56.1)	45.49±21.01	23.25(20.87,39.75)	-0.85	-2.01	-2.30	0.398	0.044	0.022
**PD-1^+^CD56^dim^**	1.97(0.964, 3.9)	1.83(0.9,2.78)	0.72(0.29,1.73)	-0.97	-2.35	-2.17	0.331	0.019	0.030	1.5(0.728, 5.16)	0.84(0.20,4.09)	0.40(0.21,1.22)	-1.52	-2.11	-1.73	0.128	0.035	0.084
**CD56^bright^CD16^-^**	3.58(1.94,5.60)	4.10(3.06,6.57)	10.70±5.95	-1.46	-3.17	-3.26	0.144	0.002	0.001	0.1164(0.0605, 0.1961)	0.0580(0.0396,0.1477)	0.0924(0.0590,0.1828)	-2.37	-0.50	-0.17	0.018	0.619	0.868
**CD69^+^CD56^bright^**	3.10(1.95, 5.07)	3.46(2.36,4.02)	1.72(0.33,4.52)	-0.25	-0.52	-0.16	0.804	0.601	0.877	2.97(2.14, 4.2)	3.41(1.92,12.17)	2.26(1.32,18.42)	-2.37	-0.45	-0.17	0.018	0.653	0.868
**CD57^+^CD56^bright^**	0.48(0.00, 1.41)	0.58(0,1.48)	0.82(0.43,1.25)	-0.75	-0.11	-1.82	0.454	0.911	0.069	0.51(0.24, 1.16)	0.60(0,1.54)	1.51(0.54,2.18)	-0.41	-2.01	-1.97	0.686	0.044	0.049
**TIGIT^+^CD56^bright^**	8.10(5.79, 12.3)	9.57(6.84,16.06)	6.28(3.43,11.16)	-0.77	-1.20	-1.76	0.443	0.232	0.079	9.04(6.72, 14.8)	8.26(5.66,19.43)	5.34(1.55,13.55)	-0.17	-1.59	-1.73	0.866	0.113	0.084
**PD-1^+^CD56^bright^**	0.24(0.00, 0.79)	0.00(0.00,0.3)	0.00(0.00,0.36)	-1.68	-1.70	-1.53	0.094	0.088	0.126	0.033(0, 0.991)	0(0, 0.34)	0(0,0.41)	-0.37	-0.09	-0.16	0.715	0.925	0.875

### Correlation between NK phenotype and clinical indicators

In the initial treatment group, HBV DNA was positively correlated with CD69^+^CD56^dim^ (β = -0.35, t = -2.27, *P* = 0.029) and on the other hand, had negative correlation with CD56^bright^ CD16^-^ (β = 0.43, t = 2.15, *P* = 0.038), as shown in **Table 3**.

**Table 3:** Correlation between NK phenotype and clinical indicators.

	Initial Treatment Group				Plateau Group				NA			
	CD56dim	CD56dimCD69	CD56dimCD57	CD56bright	CD56dim	CD56dimCD69	CD56dimCD57	CD56bright	CD56dim	CD56dimCD69	CD56dimCD57	CD56bright
HBsAg	t=-0.144 P=0.886	t=0.426 P=0.672	t=1.251 P=0.218	t=0.818 P=0.418	t=-1.168 P=0.249	t=0.209 P=0.836	t=-0.181 P=0.858	t=-0.832 P=0.41	t=-0.546 P=0.59	t=-0.425 P=0.675	t=-0.933 P=0.36	t=-0.744 P=0.464
HBeAg	t=1.164 P=0.251	t=-1.253 P=0.218	t=1.192 P=0.24	t=2.34 P=0.024	t=-0.051 P=0.96	t=-0.712 P=0.481	t=-0.525 P=0.602	t=0.071 P=0.944	t=-0.486 P=0.631	t=0.958 P=0.346	t=-0.092 P=0.928	t=-0.198 P=0.845
HBV DNA	t=0.209 P=0.835	t=-2.273 P=0.029	t=1.326 P=0.193	t=2.153 P=0.038	t=-1.757 P=0.087	t=0.976 P=0.335	t=-0.459 P=0.649	t=-0.849 P=0.401	t=0.079 P=0.937	t=0.006 P=0.995	t=-1.208 P=0.238	t=1.316 P=0.2
ALT	t=-0.057 P=0.955	t=-0.5 P=0.62	t=1.683 P=0.102	t=1.563 P=0.128	t=-0.704 P=0.486	t=0.285 P=0.777	t=-1.143 P=0.26	t=-0.872 P=0.388	t=0.125 P=0.901	t=-1.149 P=0.261	t=-0.581 P=0.566	t=-0.442 P=0.662
AST	t=-0.314 P=0.756	t=-0.366 P=0.717	t=1.7 P=0.099	t=1.515 P=0.14	t=-0.528 P=0.6	t=0.373 P=0.711	t=-1.208 P=0.235	t=-0.796 P=0.431	t=0.013 P=0.99	t=-1.201 P=0.24	t=0.247 P=0.807	t=0.872 P=0.391

## Discussion

In the process of antiviral treatment for CHB, the therapeutic effect is closely related to the virus type, treatment strategy and immune status (16). During the use of IFN, our previous research found that 75% of the disappearance of HBsAg occurred after 48 weeks, and prolonged treatment was helpful for the disappearance of HBsAg and maintenance of antiviral effect (17). However, not all patients can obtain the ideal effect through prolonged treatment, and the decline of HBsAg in some patients stagnates with the extension of treatment time. Based on this phenomenon, we have conducted clinical studies and found that about 20% of patients achieved the disappearance of surface antigens through the intermittent treatment mode of initial - intermittent - retreat, which proved the effectiveness of this strategy (8). But the molecular mechanisms behind intermittent therapy, and why platform of HBsAg level occurs, remain to be explored. In this study, by observing the changes of NK cell-related phenotypes in patients at different treatment stages in different times, we explored the molecular mechanism of the emergence of the plateau, providing a theoretical basis for intermittent therapy.

NK cells, as a component of innate immune cells with significant importance in the human body, and play a role in eliminating viruses through cytotoxicity or secretion of cytokines which can induce inflammation reaction (18). Chronic HBV infection results in decreased NK cell count and function. IFN, as an antiviral drug recommended by the guidelines, induces ISG transcription through the JAK/STAT pathway and targets to interfere with different stages of viral replication, including virus invasion, uncoating, gene recombination, and assembly machine release, acting as a resistance power in viral infection (15, 19–21). Interferon has been reported to significantly amplify CD56^bright^CD16^-^ cells and promote the activation of CD56^dim^CD16^+^ cells (22). CD56^dim^CD16^+^ and CD56^bright^CD16^-^ are the main subgroups of NK cells, and CD56^dim^CD16^+^ plays the key role of killing, accounting for about 90% of NK cells, and exerts cytotoxic effect through the secretion of granulozyme and perforin. CD56^bright^CD16^-^ mainly works with regulatory function, accounting for about 10% of the total NK cells, and secretes TNF-α, IFN-γ and other cytokines (23). In this study, in addition to the determination of CD56^bright^CD16^-^ and CD56^bright^CD16^-^ subgroups, we also continued to gate the two subgroups to detect the expressions of CD69, CD57, TIGIT and PD-1 in the two subgroups respectively. CD69 is an activating molecule of NK cells and a killer receptor (24, 25). CD57 is expressed in mature and differentiated NK cells, marking the maturity of NK cells (26). Both TIGIT and PD-1 are co-inhibitory receptors, which are specifically expressed on the surface of NK cells and T cells, playing a co-inhibitory role. Studies have reported that TIGIT acts on liver cytotoxic T lymphocyte (CTL), leading to immune tolerance and making HBV difficult to clear. Blocking TIGIT pathway or inhibiting TIGIT expression can restore immune function (27, 28). In an animal experiment, PD-1 deficient mice had an advantage in liver clearance of adenovirus, but they were also prone to immune damage, which was speculated to be due to the excessive autoimmune reaction caused by PD-1 deficiency (29). Therefore, the moderate expression of TIGIT and PD-1 is also extremely important in innate immunity.

Our research found that patients treated with IFN for a period of time had significant differences in the percentage of CD56^dim^CD16^+^, CD69^+^CD56^dim^, and CD57^+^CD56^dim^ compared with the initial treatment group. The expression of CD56^dim^CD16^+^ and CD57^+^CD56^dim^ decreased in patients treated with IFN, indicating that the subgroup which played a killing role was weakened and the number of mature cells decreased after IFN treatment for a period of time. The frequency of CD69^+^CD56^dim^ increased, indicating that although the killing subsets decreased, the activity of NK cells in the killing subsets increased in patients treated with IFN. Studies have shown that the elevation of transaminase during interferon trial is associated with the upregulation of NKCD69. This study can further confirm that in patients treated with IFN, the killing subgroup CD69^+^CD56^dim^ is significantly up-regulated, which enhances the killing activity and may cause abnormal transaminase. During the follow-up of patients initially treated with IFN, the frequency of CD56^dim^CD16^+^ gradually decreased with the extension of IFN use time, indicating that the killing function of NK cells was gradually weakened with the application of IFN, which may lead to the emergence of a plateau period. At the same time, the frequency of CD56^bright^CD16^-^ increased gradually, because the differentiation direction of NK cells was from regulated subgroup to killing subgroup, that is, from CD56^bright^CD16^-^ to CD56^dim^CD16^+^, and finally to exhaustion. Therefore, it can be considered that the change of CD56^bright^CD16^-^ frequency is due to the compensatory increase due to the decrease of CD56^dim^.

It has been shown that HBsAg decline of less than 0.5lgIU/ml from baseline after 12 weeks of PEG-IFN treatment is indicative of difficulty in achieving sustained virological remission after cessation of treatment (30). This suggests that if a significant and sustained decline of HBsAg does not occur during treatment, it is difficult to obtain a good follow-up response. Therefore, we took the HBsAg decline range ≤ 0.5lgIU/ml as the standard of the plateau period.

In plateau group, with the extension of IFN discontinuation period, the frequency of NK cell-related subsets had gradually recovered. The frequency of CD57^+^CD56^dim^ increased significantly after 12-24 weeks of IFN suspension compared to baseline, indicating the maturation of the killing subgroup. Compared with CHB patients who had not received any antiviral drugs (baseline in the initial treatment group), the frequency of CD57^+^CD56^dim^ returned to the ideal level after 12 to 24 weeks of discontinuation of IFN during the plateau period. However, the frequency of some subgroups is still different from that of untreated patients, which needs to be further discussed with additional follow-up data.

Studies have shown that reducing viral load through antiviral therapy can partially reshape the function of NK cells. Reducing viral load is beneficial to improve NK cell function (31). In pregnant women infected with HBV, antiviral therapy can significantly increase the frequency of NK cells (32). In this study, through multiple linear regression analysis of baseline clinical indicators and NK subpopulation rows, it was found that the HBV DNA level in the initial treatment group was negatively correlated with the frequency of CD69^+^CD56^dim^, suggesting that viral load may inhibit the activation of NK cells.

In conclusion, this study increased the difference in the expression of NK cell subsets in patients without any treatment and IFN-treated, and provided part of the theoretical basis for the emergence of a plateau in IFN treatment. From the perspective of NK cells, it provides theoretical support for intermittent treatment of interferon. The results showed that during IFN treatment, the killing subsets of NK cells decreased gradually, the compensatory subsets increased, and the activity of killing subsets increased. After interferon was discontinued, the frequency and function of NK cell subsets gradually recovered. High virus levels affect the function of NK cells and affect the activation of NK cells.

It is recommended according to the guideline that treatment modification should not be considered until the desired results were not achieved after 24 weeks of interferon therapy (1). For the initial treatment group, our follow-up time nodes were 4 weeks and 12-24 weeks, respectively. During the 24 weeks, we will not consider changing the treatment regimen, so no patients from the initial treatment group will enter the platform group. However, with the extension of follow-up time, it is not excluded that some patients in the initial treatment group will enter the platform group due to the stagnation of surface antigen decline. This is also the limitation of this study. We will continue to follow up and focus on those patients who entered the platform group from the initial treatment group.

As a novel serological marker, HBV RNA contains HBV pre-genomic RNA and its shear variants, which can reflect the activity of hepatic cccDNA to a certain extent (33). Therefore, it is also of great significance in reflecting the response to antiviral therapy and predicting relapse after drug withdrawal. However, a number of recent studies have confirmed that HBV RNA cannot be used as an indicator of drug withdrawal in patients with CHB (34, 35). Compared with classical serological indicators such as HBsAg, HBV DNA, ALT, etc., the clinical guiding significance of HBV RNA still needs to be studied.

Interferon-gamma-induced protein10 (IP-10), also known as C-X-C ligand 10 (CXCL10), belongs to the CXC family of chemokines and plays an important role in the pathogenesis of immune diseases and inflammatory diseases. Serum IP-10 expression levels were higher in patients with higher levels of liver inflammation and fibrosis (36). In addition, intrahepatic IP-10 levels were positively correlated with oral efficacy in patients with CHB (37). However, its response to the degree of fibrosis and prediction of antiviral efficacy are also correlated with other clinical indicators, such as ALT, AST, HBV DNA, and HBsAg levels. There is currently a lack of large sample size studies to confirm the relationship between IP-10 and antiviral efficacy. Compared with classical clinical indicators such as HBV DNA, IP-10, as an emerging clinical indicator, needs further research. In summary, we did not conduct correlation analysis of HBV RNA, IP-10 and NK cell phenotypes in this study. However, in subsequent studies, we will include more clinical indicators and more cytokines for analysis.

The analysis of clinical characteristic in plateau group at the beginning use of IFN was in absence. We have studied in our previous article that HBsAg response at 12 weeks and 24 weeks of initial treatment was significantly correlated with the effect of retreatment after plateau. Compared with patients who did not respond to initial treatment, patients who developed an antiviral response early in treatment had a higher rate of response after intermittent treatment during the plateau period. In the platform group, HBsAg level at first use of IFN, early treatment response, HBsAg level at discontinuation of IFN, and duration of IFN use were all significantly correlated with functional recovery by intermittent therapy (8). Moreover, in this study, with the extension of follow-up time, some patients in the initial treatment group will be transferred to the plateau group later, and their data is more comprehensive and representative. We have been working on the follow-up work, and we believe it will be elaborated in a future article.

There are also several limitations in this research, such as small sample size, lack of whole period data from initial treatment to plateau stage. Besides, counting NK cell subsets can more accurately explain the effect of interferon treatment on NK cells. Therefore, more evidence and work are need for the further exploration.

## Data availability statement

The raw data supporting the conclusions of this article will be made available by the authors, without undue reservation.

## Ethics statement

The studies involving human participants were reviewed and approved by Ethics Committee of Beijing Ditan Hospital (Jing Di Lun Ke Zi 2018 no. 023-01). The patients/participants provided their written informed consent to participate in this study.

## Author contributions

ML and YX contributed to study concept and design. MC, SX and LY conducted experiments and collected the data. YLu, GS, SX and LY collected the information of the patients. TJ, WD, SWa, LZ, YLi and YG provided reagents to help the experimental work. YLi analyzed the statistical results. HH, SWu, RL, MX, LH and XC edited the English version. All authors contributed to the article and approved the submitted version.

## References

[1] Chinese Society of Infectious Diseases CMA Chinese Society of Hepatology CMA. The guidelines of prevention and treatment for chronic hepatitis b (2019 version). Zhonghua Gan Zang Bing Za Zhi (2019) 27(12):938–61. doi: 10.3760/cma.j.issn.1007-3418.2019.12.007 PMC12813922 31941257

[2] Tseng TC Liu CJ Yang HC Su TH Wang CC Chen CL. Determinants of spontaneous surface antigen loss in hepatitis b e antigen-negative patients with a low viral load. Hepatology (2012) 55(1):68–76. doi: 10.1002/hep.24615 21858846

[3] Yip TC Wong GL Chan HL Tse YK Lam KL Lui GC. Hbsag seroclearance further reduces hepatocellular carcinoma risk after complete viral suppression with Nucleos(T)Ide analogues. J Hepatol (2019) 70(3):361–70. doi: 10.1016/j.jhep.2018.10.014 30367899

[4] Yeo YH Ho HJ Yang HI Tseng TC Hosaka T Trinh HN. Factors associated with rates of hbsag seroclearance in adults with chronic hbv infection: A systematic review and meta-analysis. Gastroenterology (2019) 156(3):635–46 e9. doi: 10.1053/j.gastro.2018.10.027 30342034

[5] Li MH Zhang L Qu XJ Lu Y Shen G Wu SL. Kinetics of hepatitis b surface antigen level in chronic hepatitis b patients who achieved hepatitis b surface antigen loss during pegylated interferon alpha-2a treatment. Chin Med J (Engl) (2017) 130(5):559–65. doi: 10.4103/0366-6999.200554 PMC5339929 28229987

[6] European Association for the Study of the Liver. Electronic address eee, European association for the study of the l. easl 2017 clinical practice guidelines on the management of hepatitis b virus infection. J Hepatol (2017) 67(2):370–98. doi: 10.1016/j.jhep.2017.03.021 28427875

[7] Zhang WH Zhang DZ Dou XG Xie Q Jiang JJ Chen XY. Consensus on pegylated interferon alpha in treatment of chronic hepatitis b. Zhonghua Gan Zang Bing Za Zhi (2017) 25(9):678–86. doi: 10.3760/cma.j.issn.1007-3418.2017.09.007 PMC12769108 29108190

[8] Li M Xie S Bi X Sun F Zeng Z Deng W. An optimized mode of interferon intermittent therapy help improve hbsag disappearance in chronic hepatitis b patients. Front Microbiol (2022) 13:960589. doi: 10.3389/fmicb.2022.960589 36110295 PMC9468551

[9] Lam VC Lanier LL. Nk cells in host responses to viral infections. Curr Opin Immunol (2017) 44:43–51. doi: 10.1016/j.coi.2016.11.003 27984782 PMC5451301

[10] Schuch A Hoh A Thimme R. The role of natural killer cells and Cd8(+) T cells in hepatitis b virus infection. Front Immunol (2014) 5:258. doi: 10.3389/fimmu.2014.00258 24917866 PMC4042360

[11] Zimmer CL Rinker F Honer Zu Siederdissen C Manns MP Wedemeyer H Cornberg M. Increased nk cell function after cessation of long-term Nucleos(T)Ide analogue treatment in chronic hepatitis b is associated with liver damage and hbsag loss. J Infect Dis (2018) 217(10):1656–66. doi: 10.1093/infdis/jiy097 29471497

[12] Edlich B Ahlenstiel G Zabaleta Azpiroz A Stoltzfus J Noureddin M Serti E. Early changes in interferon signaling define natural killer cell response and refractoriness to interferon-based therapy of hepatitis c patients. Hepatology (2012) 55(1):39–48. doi: 10.1002/hep.24628 21898483 PMC3353526

[13] Ahlenstiel G Titerence RH Koh C Edlich B Feld JJ Rotman Y. Natural killer cells are polarized toward cytotoxicity in chronic hepatitis c in an interferon-Alfa-Dependent manner. Gastroenterology (2010) 138(1):325–35 e1-2. doi: 10.1053/j.gastro.2009.08.066 19747917 PMC2862622

[14] Cao W Lu H Zhang L Wang S Deng W Jiang T. Functional molecular expression of nature killer cells correlated to hbsag clearance in hbeag-positive chronic hepatitis b patients during peg-ifn A-2a therapy. Front Immunol (2022) 13:1067362. doi: 10.3389/fimmu.2022.1067362 36479104 PMC9720173

[15] Schneider WM Chevillotte MD Rice CM. Interferon-stimulated genes: A complex web of host defenses. Annu Rev Immunol (2014) 32:513–45. doi: 10.1146/annurev-immunol-032713-120231 PMC4313732 24555472

[16] Wang J Du L Tang H. Suppression of interferon-alpha treatment response by host negative factors in hepatitis b virus infection. Front Med (Lausanne) (2021) 8:784172. doi: 10.3389/fmed.2021.784172 34901094 PMC8651562

[17] Li M Zhang L Lu Y Chen Q Lu H Sun F. Early serum hbsag kinetics as predictor of hbsag loss in patients with hbeag-negative chronic hepatitis b after treatment with pegylated interferonalpha-2a. Virol Sin (2021) 36(2):311–20. doi: 10.1007/s12250-020-00290-7 PMC8087759 32975731

[18] Paolini R Bernardini G Molfetta R Santoni A. Nk cells and interferons. Cytokine Growth Factor Rev (2015) 26(2):113–20. doi: 10.1016/j.cytogfr.2014.11.003 25443799

[19] Brunetto MR Marcellin P Cherubini B Yurdaydin C Farci P Hadziyannis SJ. Response to peginterferon Alfa-2a (40kd) in hbeag-negative chb: On-treatment kinetics of hbsag serum levels vary by hbv genotype. J Hepatol (2013) 59(6):1153–9. doi: 10.1016/j.jhep.2013.07.017 23872601

[20] Sajid M Liu L Sun C. The dynamic role of nk cells in liver cancers: Role in hcc and hbv associated hcc and its therapeutic implications. Front Immunol (2022) 13:887186. doi: 10.3389/fimmu.2022.887186 35669776 PMC9165341

[21] Anggakusuma Romero-Brey I Berger C Colpitts CC Boldanova T Engelmann M. Interferon-inducible cholesterol-25-Hydroxylase restricts hepatitis c virus replication through blockage of membranous web formation. Hepatology (2015) 62(3):702–14. doi: 10.1002/hep.27913 25999047

[22] Xu D Fu J Jin L Zhang H Zhou C Zou Z. Circulating and liver resident Cd4+Cd25+ regulatory T cells actively influence the antiviral immune response and disease progression in patients with hepatitis b. J Immunol (2006) 177(1):739–47. doi: 10.4049/jimmunol.177.1.739 16785573

[23] Mondelli MU Varchetta S Oliviero B. Natural killer cells in viral hepatitis: Facts and controversies. Eur J Clin Invest (2010) 40(9):851–63. doi: 10.1111/j.1365-2362.2010.02332.x 20597961

[24] Shiow LR Rosen DB Brdickova N Xu Y An J Lanier LL. Cd69 acts downstream of interferon-Alpha/Beta to inhibit S1p1 and lymphocyte egress from lymphoid organs. Nature (2006) 440(7083):540–4. doi: 10.1038/nature04606 16525420

[25] Stegmann KA Bjorkstrom NK Veber H Ciesek S Riese P Wiegand J. Interferon-Alpha-Induced trail on natural killer cells is associated with control of hepatitis c virus infection. Gastroenterology (2010) 138(5):1885–97. doi: 10.1053/j.gastro.2010.01.051 20334827

[26] Bjorkstrom NK Riese P Heuts F Andersson S Fauriat C Ivarsson MA. Expression patterns of Nkg2a, kir, and Cd57 define a process of Cd56dim nk-cell differentiation uncoupled from nk-cell education. Blood (2010) 116(19):3853–64. doi: 10.1182/blood-2010-04-281675 20696944

[27] Joller N Hafler JP Brynedal B Kassam N Spoerl S Levin SD. Cutting edge: Tigit has T cell-intrinsic inhibitory functions. J Immunol (2011) 186(3):1338–42. doi: 10.4049/jimmunol.1003081 PMC3128994 21199897

[28] Johnston RJ Comps-Agrar L Hackney J Yu X Huseni M Yang Y. The immunoreceptor tigit regulates antitumor and antiviral Cd8(+) T cell effector function. Cancer Cell (2014) 26(6):923–37. doi: 10.1016/j.ccell.2014.10.018 25465800

[29] Zhang Z Zhang JY Wherry EJ Jin B Xu B Zou ZS. Dynamic programmed death 1 expression by virus-specific Cd8 T cells correlates with the outcome of acute hepatitis b. Gastroenterology (2008) 134(7):1938–49, 49 e1-3. doi: 10.1053/j.gastro.2008.03.037 18455515

[30] Moucari R Mackiewicz V Lada O Ripault MP Castelnau C Martinot-Peignoux M. Early serum hbsag drop: A strong predictor of sustained virological response to pegylated interferon Alfa-2a in hbeag-negative patients. Hepatology (2009) 49(4):1151–7. doi: 10.1002/hep.22744 19115222

[31] Cao W Li M Zhang L Lu Y Wu S Shen G. The characteristics of natural killer cells in chronic hepatitis b patients who received pegylated-interferon versus entecavir therapy. BioMed Res Int (2021) 2021:2178143. doi: 10.1155/2021/2178143 33575322 PMC7857883

[32] Wang F Xie S Ran C Hao H Jiang T Deng W. Effect of antiviral therapy during pregnancy on natural killer cells in pregnant women with chronic hbv infection. Front Immunol (2022) 13:893628. doi: 10.3389/fimmu.2022.893628 35677040 PMC9168030

[33] Butler EK Gersch J McNamara A Luk KC Holzmayer V de Medina M. Hepatitis b virus serum DNA andrna levels in Nucleos(T)Ide analog-treated or untreated patients during chronic and acute infection. Hepatology (2018) 68(6):2106–17. doi: 10.1002/hep.30082 29734472

[34] Fan R Zhou B Xu M Tan D Niu J Wang H. Association between negative results from tests for hbv DNA and rna and durability of response after discontinuation of Nucles(T)Ide analogue therapy. Clin Gastroenterol Hepatol (2020) 18(3):719–27 e7. doi: 10.1016/j.cgh.2019.07.046 31362119

[35] Kaewdech A Tangkijvanich P Sripongpun P Witeerungrot T Jandee S Tanaka Y. Hepatitis b surface antigen, core-related antigen and hbv rna: Predicting clinical relapse after Na therapy discontinuation. Liver Int (2020) 40(12):2961–71. doi: 10.1111/liv.14606 32668074

[36] Sonneveld MJ Arends P Boonstra A Hansen BE Janssen HL. Serum levels of interferon-Gamma-Inducible protein 10 and response to peginterferon therapy in hbeag-positive chronic hepatitis b. J Hepatol (2013) 58(5):898–903. doi: 10.1016/j.jhep.2013.01.029 23376362

[37] Jaroszewicz J Ho H Markova A Deterding K Wursthorn K Schulz S. Hepatitis b surface antigen (Hbsag) decrease and serum interferon-inducible protein-10 levels as predictive markers for hbsag loss during treatment with Nucleoside/Nucleotide analogues. Antivir Ther (2011) 16(6):915–24. doi: 10.3851/IMP1866 21900724

